# Examining the impact of a restorative breath-based intervention “*Sudarshan Kriya Yoga*” at work: a field experiment

**DOI:** 10.3389/fpsyg.2024.1327119

**Published:** 2024-03-05

**Authors:** Raina Chhajer, Chirag Dagar

**Affiliations:** ^1^Humanities and Social Sciences Area, Indian Institute of Management, Indore, India; ^2^XLRI - Xavier School of Management, Jamshedpur, India

**Keywords:** *Sudarshan Kriya Yoga* (SKY), breath-based interventions, workplace, stress, anxiety, well-being, health, thriving

## Abstract

**Background:**

Human capital plays a crucial role in the success of an organization and further contributes to the broader goals of growth and development of society. In this regard, it is essential to ensure the well-being of employees at the workplace. Given the positive impact of yoga on psycho-physiological aspects of health, this study aims to examine the impact of a breath-based yogic intervention, *Sudarshan Kriya Yoga* (SKY), on stress, anxiety, thriving, general health, emotional well-being, social well-being, and psychological well-being among employees of a leading manufacturing firm in India.

**Methods:**

Undertaking a randomized-control experiment design (*n* = 64), we examined the impact of SKY on stress, anxiety, thriving, general health, and emotional, social, and psychological well-being. Two certified instructors conducted the SKY intervention in a retreat format over 3 days.

**Results:**

The analysis demonstrated positive outcomes across various aspects of participants’ well-being, i.e., it significantly reduced their stress and anxiety and increased the levels of thriving, general health, and emotional, social, and psychological well-being. These findings are valuable for understanding the potential benefits of the SKY intervention.

**Discussion:**

The findings provide support for considering SKY as a potential well-being intervention for employers at the workplace and society at large. Further exploration, implementation, and research in diverse contexts will be crucial to fully understand the long-term impact and scalability of the SKY intervention in promoting holistic well-being.

## Introduction

Yoga is a holistic discipline and way of life originating from ancient India that promotes relaxation and a harmonious mental state and aims to integrate various aspects of an individual, including the physical, vital, mental, and spiritual dimensions (Nosaka and Okamura, 2015). Sage *Patanjali* described the yogic philosophy and practice in the classic text, *YogaSutras*, and outlined the eight-limbed path of yoga. The eight-limbed structure, i.e., *Ashtanga* yoga, comprises *yamas* (abstentions), *niyamas* (observances), *asanas* (postures), *praṇayama* (control of breath), *pratyahara* (withdrawal of senses), *dharaṇa* (concentration), *dhyana* (meditation), and *samadhi* (oneness) (Novaes et al., 2020). Although people today identify yoga only with *asana*, denoting the physical practice of yoga, it represents one of the many tools aimed at self-transformation (Woodyard, 2011). Notably, in contemporary times, the common practice of yoga involves a combination of physical postures, breathing practices, and meditation (Collins, 1998).

The body of knowledge supporting the positive impact of yoga on health and well-being across diverse populations is expanding (see Evans et al., 2009; Hagen and Nayar, 2014; Park et al., 2015). Research indicates that yoga enhances positive emotions while reducing negative emotions (Narasimhan et al., 2011; Felver et al., 2015). Studies have also demonstrated that yoga helps mitigate stress (Gard et al., 2012; Rocha et al., 2012) and improve psychological states associated with high-stress levels, including excessive worry or anxiety (Li and Goldsmith, 2012) and mood (Streeter et al., 2010). In the context of the workplace comprising healthcare workers, employees at university, school principals, and employees, yoga has been found to be effective in decreasing stress, anxiety, depression, and insomnia and enhancing well-being (Van der Merwe and Parsotam, 2011; Nosaka and Okamura, 2015; Maddux et al., 2018; Cocchiara et al., 2019).

*Praṇayama* comprises the Sanskrit word *prana* (vital energy) and *ayama* (control). It involves a series of voluntary controlled breathing exercises that aim at manipulating the respiration activity, inhalation (*puraka*), retention (*kumbhaka*), exhalation (*rechaka*), and body locks (*bandhas*) (Novaes et al., 2020). Encompassing the breathing practices, which are intrinsic to *pranayama*, is the control and augmentation of breath, which is postulated as the bridge between the body and mind (Ankad et al., 2011). Sage *Patanjali* has ascribed *pranayama* with greater significance in *Ashtanga* yoga, even more than *asanas*, for keeping sound health (Sharma et al., 2013).

*Pranayama* techniques have demonstrated notable effects on both human physiology and psychology. The three processes of inhalation, retention, and exhalation can be either fast or slow, whereby research indicates that both fast and slow *pranayamas* are beneficial for sustained attention and the ability to shift attention and cardiac functions of the body (Udupa et al., 2003; Telles et al., 2008). The positive impact of *pranayama* on neurocognitive abilities has also been highlighted with enhanced performance on spatial and verbal memory tasks, auditory and visual reaction time, and other executive functions (Saoji et al., 2019). Research utilizing *pranayama* practices as interventions has highlighted a decrease in hypertension, respiratory rate, and stress levels, along with an increase in heart rate variability and the balance between sympathetic and parasympathetic nervous systems, among others (Peterson et al., 2017). Emerging research has also reported the efficacy of breathing practices in improving perceived stress, anxiety, negative affect, sleep, social connectedness, self-esteem, life esteem, and general well-being (Kamath et al., 2017; Novaes et al., 2020; Goldstein et al., 2022).

Extant literature indicates that mind–body interventions, including yoga and other meditation programs, render potential solutions in the form of tools and techniques to reduce stress and improve work-related outcomes such as greater productivity, reduced turnover, job satisfaction, and engagement at work (Jacobs and Blustein, 2008; Shiba et al., 2015; Axén and Follin, 2017). Importantly, owing to the vitality, creativity, and productivity it entails, the human capital of a society denotes an instrumental resource directly affecting the sustenance and development of businesses and impacting the growth, development, and social stability of nations (Hendricks, 2002; Wright and McMahan, 2011). However, in current work organizations, delivering extraordinary performance outcomes, always needing to be accessible, and working for long durations have become a norm (Von Bergen and Bressler, 2019). Literature indicates that higher individual initiatives are associated with higher work overload, job stress, and work–family conflicts, adversely impacting employee health, well-being, and performance (Bolino and Turnley, 2005). Consequently, work-related stress and burnout have been found to incur organizational costs, including decreased productivity, high employee turnover, and absenteeism (Garton, 2017; Hassard et al., 2018).

Given the adverse trickle-down effect of burnout and stress, implementing strength-oriented initiatives and targeting well-being has become imperative at the workplace. The World Health Organization (WHO) suggests that workplaces can be key to health promotion (Jia et al., 2018). As a result, to combat these challenges, organizations, including Google, Intel, and IBM, have introduced mindfulness, yoga, and breathing-based programs, such as “Search Inside Yourself,” “Awake,” and “Mindfulness,” respectively, for their employees (Schaufenbuel, 2014, 2015; Gupta and Singh, 2019). Such programs in the workplace have seen significant growth wherein these have been deployed in the occupational setup and are not limited to addressing psychological concerns among employees but are being offered to the general workforce, and have shown positive results on outcomes such as distress, depression, burnout, health, job performance, compassion, empathy, and well-being (Lomas et al., 2019). In 2018, approximately 60% of mid-sized to large American companies disclosed that they provide their employees with access to courses in mindfulness, yoga, or meditation (Vonderlin et al., 2020). These include a broad array of sectors, including but not limited to technology firms (e.g., Microsoft and Apple), social media (e.g., Facebook and LinkedIn), industrial firms (e.g., Beiersdorf and Bosch), apparel and furniture (e.g., Nike and IKEA), financial and insurance service providers (e.g., Goldman Sachs), as well as political institutions. It has been found that these programs successfully alleviate stress, mitigate burnout, reduce mental distress and somatic complaints, and concurrently enhance mindfulness, well-being, and job satisfaction (Vonderlin et al., 2020).

Despite the growth in the study of *pranayama*, there still needs to be more understanding of the effectiveness of different breathing techniques (Novaes et al., 2020; Goldstein et al., 2022). While some organizations are adopting similar approaches, there is a need to implement more such mind–body-based restorative breathing interventions at work (Gupta and Singh, 2019). As a result, drawing on the body of knowledge representing yoga and *pranayama*, the current study looks at the breath-based intervention of SKY. SKY is a distinctive (rhythmic) yogic breathing technique designed and taught by the Art of Living Foundation, an international not-for-profit organization founded in 1981 by Sri Sri Ravi Shankar (Agte and Chiplonkar, 2008). This practice’s gentle and distinct rhythm infuses each body cell with oxygen and *prana* (breath), eliminating both physical and emotional toxins (Pandya, 2014). Consistent practice contributes to the development of intuition, the sharpening of intellect, improved memory, heightened clarity, stress reduction, and the flourishing of creativity (Pandya, 2014).

Compelling evidence in the literature indicates that SKY is beneficial and offers a low-risk alternative practice for enhancing overall health and well-being (Sureka et al., 2014; Seppälä et al., 2020). Studies have demonstrated the significant positive effects of SKY concerning mental disorders, including post-traumatic stress disorder, depression, anxiety, and substance abuse among clinical populations (Zope and Zope, 2013; Seppälä et al., 2014). Additionally, SKY has been found to be effective in reducing anxiety, depression, and stress while increasing optimism among college students (Kjellgren et al., 2007). Emerging research from the workplace has also been affirming. In a sample from the border road organization, SKY was effective in stress regulation and consequently improved their workload tolerance capacity (Chandra et al., 2017). Furthermore, as a result of practicing SKY, healthcare professionals experienced a significant improvement in professional fulfillment, work exhaustion, and positivity (Kanchibhotla et al., 2022). Research conducted with participants from a technology, engineering, construction, and manufacturing organization reported stress reduction, increased life satisfaction, enhanced emotional stability, and improved emotional regulation among participants (Mulla and Vedamuthachar., 2014). Senior government officers reported similar positive impacts for better sleep quality, trauma reduction, cortisol regulation, and anger management (Kolhe, 2022).

While the literature supports the effectiveness of SKY in clinical populations (Zope and Zope, 2013), there is a need for additional research, especially in non-clinical contexts such as work organizations (Mulla and Vedamuthachar, 2014; Kolhe, 2022). Recognizing that the absence of psychological issues does not necessarily equate to optimal well-being (Keyes, 2007), through this study, we explore the effectiveness of a comprehensive breath-based SKY intervention and its ability to reduce stress and anxiety and enhance employees’ well-being.

Stress at work is defined as “a situation wherein job-related factors interact with the worker to change his or her psychological and/or physiological conditions such that the person is forced to deviate from normal functioning” (Newman and Beehr, 1979). Research shows that job stress impairs employees’ health, well-being, and performance (Kotteeswari and Sharief, 2014). Burnout is conceptualized in the context of stress at work as a consequence of being exposed to chronic work-related stressors (Hobfoll and Shirom, 2000). It is associated with job dissatisfaction, turnover intentions, and physical and emotional symptoms (Pines and Keinan, 2005).

Anxiety at work has been conceptualized as comprising of “(a) dispositional workplace anxiety, i.e., individual differences in levels of nervousness and tension about job performance, and (b) situational workplace anxiety, i.e., a transient emotional state of nervousness and tension about specific job performance episodes” (Cheng and McCarthy, 2018). Anxious individuals exhibit self-doubt and low confidence and find it challenging to manage untoward situations (Shell and Husman, 2008). Existing studies have essentially rendered a negative relationship between anxiety and performance, including selection tests (Proost et al., 2008) and job interviews (McCarthy and Goffin, 2004). Scholars point out that anxiety should be driven out for optimum functioning in organizations (Reio and Callahan, 2004).

Thriving at work involves two critical dimensions of psychological experience: vitality (affective) and learning (cognitive) (Spreitzer et al., 2005; Spreitzer and Porath, 2014). Thriving individuals have a joint experience of energy and a feeling of continuous improvement at work. Research indicates that energy management enables a thriving experience (Spreitzer and Porath, 2014).

Mental health holds an essential place in the occupational psychology literature. It is examined with respect to two perspectives. First, positive mental health encompasses behaviors, attitudes, and feelings associated with personal success and satisfaction. The second perspective denotes mental health as an absence of mental illness (Banks et al., 1980). In order to be comprehensive in measuring mental health, both views need adequate examination. The general health questionnaire (Goldberg and Williams, 1988) enables the measurement of common mental health problems and the detection of minor psychiatric morbidity (Jackson, 2007).

The concept of well-being has been examined from objective perspectives, such as financial resources, education, and environmental factors, as well as subjective viewpoints, including happiness and flourishing. While some theories focus on *hedonic* or high positive and low negative emotional aspects of well-being, others emphasize *eudaimonic* aspects (i.e., meaningfulness, purpose, and good life). In contrast, others blend *hedonic* and *eudaimonic* dimensions (Ryan and Deci, 2001). As per Keyes (2002), flourishing or high levels of well-being denote a state that is not merely an absence of illnesses but encompasses complete well-being accompanied by optimal functioning. We refer to this framework in the study, wherein well-being includes high emotional (positive affect and life satisfaction), social (positive social functioning), and psychological (*eudaimonic*) well-being.

This study delves into the significance of a breath-based intervention (SKY) that extends beyond the therapeutic effects and examines its potential and promise within the context of the workplace and organizations. The primary aim of the current study is to examine the impact of a three-day SKY intervention conducted in a retreat format on stress, anxiety, thriving, general health, emotional well-being, social well-being, and psychological well-being of employees from a leading manufacturing firm in India. We hypothesize that participants who complete the breath-based intervention (SKY) will likely report significantly higher levels of thriving and general health, as well as emotional, social, and psychological well-being while reporting significantly lower stress and anxiety levels.

## Materials and methods

### Participants

In our study, participants had to meet specific inclusion criteria to be eligible. These criteria included (a) being 18 years of age or older, (b) working a minimum of 30 h per week, (c) committing to the three-day duration of the intervention, (d) not currently undergoing any other form of psychological therapy with no plans to start such therapy during the study, (e) having no previous experience with SKY or mindfulness practices, and (f) reporting no severe health issues contraindicated by SKY. Individuals meeting these criteria were considered eligible and included in the study.

Using the software G-power 3 (Faul et al., 2007), we conducted a power analysis to determine the minimum number of participants needed for the mixed-design (within-between interaction) ANOVA analysis of the study. Based on earlier studies highlighting small to large effects (Anderson et al., 1999; Kanchibhotla et al., 2022), the effect size was set at 0.25, alpha level at 0.05, and power of 0.95 (Cohen, 1992). Power analysis indicated a total sample size of 54 and a sample of 27 for each group was found to be sufficient.

**Figure 1:**
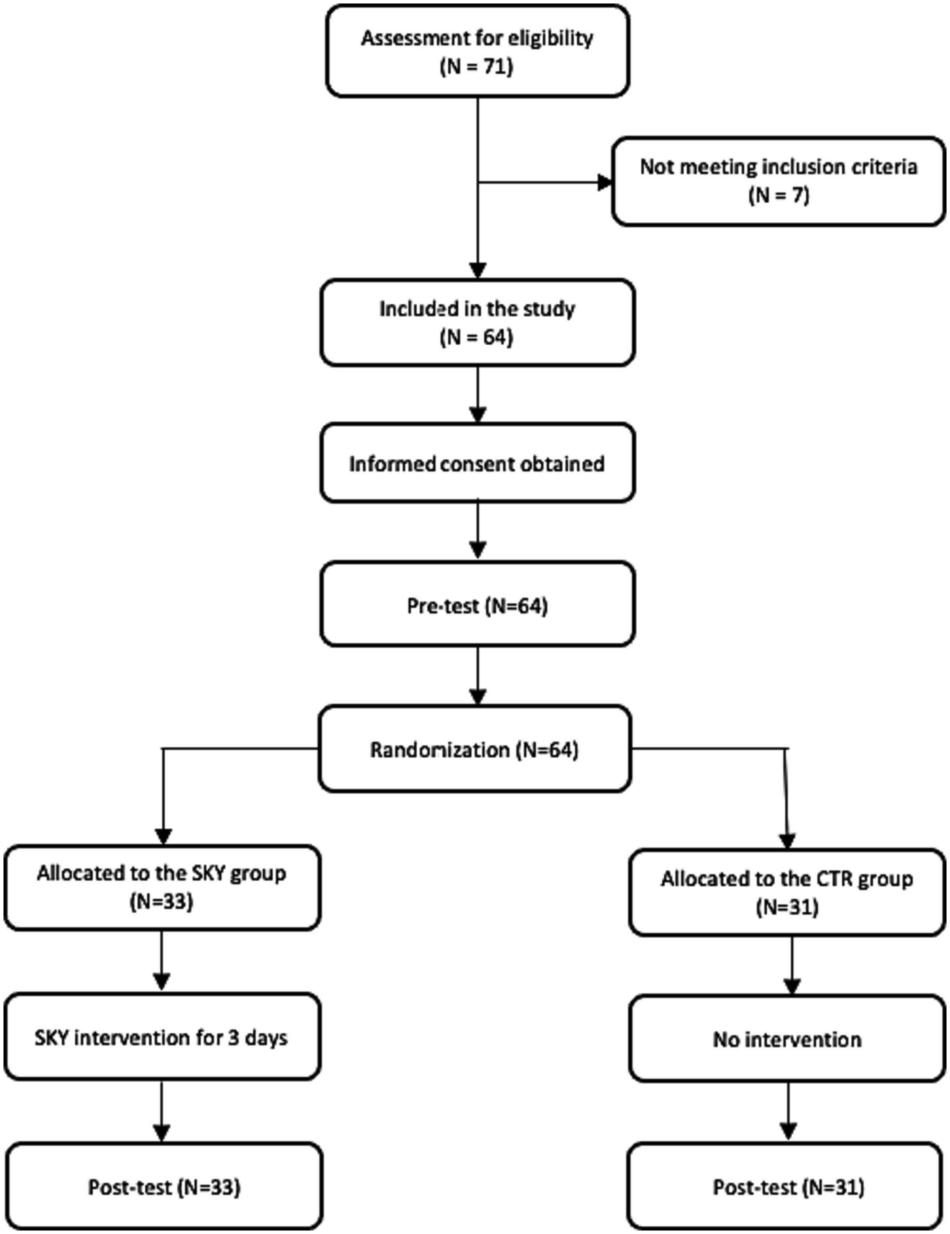
Flow chart showing the experimental design of the study.

Participants were recruited from a leading manufacturing firm in India in January 2023. Participants who worked in various departments of the organization in mid-level managerial positions were recruited via purposive sampling. Seventy one employees agreed to participate in this study. The 64 eligible participants based on the criteria mentioned earlier were randomly divided into two groups using a random number generator, with 33 employees assigned to the SKY group (SKY) and 31 to the control group (CTR). The mean age of the participants was 36 years, they were predominantly men (81.25%), and 46.87% had work experience of more than 10 years. All employees had completed graduation. Table 1 summarizes the sample characteristics, including gender, age, work experience, and education distributions. Figure 1 shows the flow chart of the experimental design of the study.

**Table 1:** Distribution of sample characteristics for SKY and control group.

Particulars	*n* = 64
**Gender**	
Female	12
Male	52
Age (years)	36 (1.5)
>30	14
31–40	31
<41	19
**Work experience (years)**	
>5	20
6–10	14
11–15	22
<16	8
**Education**	
Graduate	38
Masters	26

### Procedure

The Institutional Review Board approved the study procedure (reference number IRB/02/2022-23/HSS). Online informed consent was obtained from the participants. Before obtaining informed consent, an initial rapport was established with the participants by conducting an online interaction session. Participants were clearly informed of the voluntary nature of their participation, and assurance was provided that all collected information would be utilized solely for research purposes. Additionally, it was emphasized that findings would be presented in an aggregate manner to safeguard individual confidentiality.

Participants received an information sheet and a consent form via email before the intervention, ensuring they were well-informed about the study’s objectives and procedures. Following the data collection process, a rigorous coding procedure was implemented, with any identifiable information promptly removed to uphold participant confidentiality. These steps were undertaken to ensure a comprehensive and ethical consent process.

The participants completed the questionnaires online using Google Forms in the English language. The data collection was conducted with the help of a team from the organization’s HR department external to the study, who were blinded to group assignments. The research assistant involved in data coding was also external to the study. While complete blinding of employees was challenging as they were from the same organization, explicit instructions were provided to participants to share SKY intervention content with their peers only after its conclusion.

Also, the SKY intervention was not conducted at the workplace or during their working hours. The SKY intervention was conducted at a remote location in a large hall with enough space for participants to practice yoga on the mat. These measures were implemented to minimize bias and enhance the study’s internal validity to the extent possible given the context. Participants were asked to complete self-report measures twice: 1 week before (T1) and 1 week after (T2). The intervention was administered to the participants in the SKY group. Effects were evaluated at post-treatment. The study was conducted in accordance with the Declaration of Helsinki (as revised in 2013).

### Intervention

Participants in the SKY group attended the APEX Programme offered by the Art of Living Foundation, an international non-profit organization founded by Sri Sri Ravi Shankar. Two certified facilitators with extensive experience conducting the SKY intervention conducted the intervention in English. The intervention spanned over 3 days with an overall engagement of 24 h in a retreat format. On all 3 days, participants were engaged for 3 h in the morning and 3 h in the afternoon. The participants practiced *yoga asanas*, *ujjayi pranayama*, SKY breathwork, and guided meditation every day (see Table 2, Brown and Gerbarg, 2005; Sloan and Kanchibhotla, 2023 for details). The intervention also included a discussion component. The control group did not receive any intervention. No incentives were provided to any study participant.

**Table 2:** Overview of the SKY breathwork practice.

S. No.	Component	Content	Practice
1	*Ujjayi Pranayama*	**Victorious Breath** *Experiencing the conscious sensation of the breath touching the throat*	2–4 breaths per minute (bpm) 10 breath cycles followed by 20 s rest
2	*Bhastrika Pranayama*	**Bellows Breath** *Rapid inhalation and forceful exhalation*	15–20 bpm Three rounds followed by 20-s rest
3	*Om* Chanting	*Chanting with long expirations*	3 times Followed by 15 s rest
4	*Sudarshan Kriya*	**Correct Vision via Purifying Activity** *Rhythmic, cyclical breathing involving slow, medium, and fast breathing cycles*	Slow (20 bpm), medium (40–50 bpm), and fast (60–80 bpm) 3 repetitions of 20 slow, 40 medium, 40 rapid cycles End with 8–10 slow cycles and 5 min supine rest

### Measures

At pre-treatment and post-treatment, participants completed the following measures:

#### Stress at work

The Perceived Stress Scale, created by Cohen et al. (1983), is widely used to assess stress. In this study, four items of PSS were used. Sample items include, “*How often have you felt confident about your ability to handle your work-related problems?*.” A five-point scale is used to score responses, where 0 signifies “never” and 4 indicates “very often,” with higher scores equating to a higher stress level. The reliability calculated using Cronbach’s α was 0.44 (T1) and 0.56 (T2) in the present study.

#### Anxiety at work

We adopted seven items from the generalized anxiety scale developed by Spitzer et al. (2006). Respondents had to provide answers using a five-point Likert scale with the following categories: 1 (not at all) to 5 (extremely), with higher scores equating to a higher level of anxiety. The sample item includes, “*I am not being able to stop or control worrying.*” The reliability calculated using Cronbach’s α of anxiety measure was 0.74 (T1) and 0.77 (T2).

#### Thriving at work

We adopted the 10-item thriving at work scale developed by Porath et al. (2012). Respondents had to provide answers using a seven-point Likert scale with the following categories: 1 = strongly disagree, 2 = disagree, 3 = somewhat disagree, 4 = neutral, 5 = somewhat agree, 6 = agree, and 7 = strongly agree. The sample item includes, “*I feel alive and alert*.” The total score is obtained by summing the scores across all 10 items, with higher scores indicating greater thriving. The reliability calculated using Cronbach’s α of thriving at work measure was 0.86 (T1) and 0.87 (T2).

#### General health

We adopted 12 items from the general health questionnaire (GHQ-12) developed by Goldberg and Williams (1988). Respondents had to answer using a five-point Likert scale with the following categories: 0 (never) to 5 (very often). The sample item includes, “*I am able to concentrate.*” The total score is obtained by summing the scores across all 12 items, with higher scores indicating greater general health levels. The reliability calculated using Cronbach’s α of general health measure was 0.84 (T1) and 0.85 (T2).

#### Emotional well-being

We adopted three items from the MHC-SF scale developed by Keyes (2016) to measure emotional well-being. Respondents had to provide answers using a five-point Likert scale with the following categories: 0 (never) to 5 (every day), with higher scores equating to a higher level of emotional well-being. The sample item includes, “*I am happy at work.*” The reliability calculated using Cronbach’s α of emotional well-being measure was 0.77 (T1) and 0.75 (T2).

#### Social well-being

We adopted five items from the MHC-SF scale developed by Keyes (2016) to measure social well-being. Respondents had to provide answers using a five-point Likert scale with the following categories: 0 (never) to 5 (every day), with higher scores equating to a higher level of social well-being. The sample item includes “*That you belong to the organization.*” The reliability calculated using Cronbach’s α of social well-being measure was 0.79 (T1) and 0.77 (T2).

#### Psychological well-being

We adopted six items from the MHC-SF scale developed by Keyes (2016) to measure psychological well-being. Respondents had to provide answers using a five-point Likert scale with the following categories: 0 (never) to 5 (every day), with higher scores equating to a higher level of psychological well-being. The sample item includes, “*That you like most parts of your personality*.” The reliability calculated using Cronbach’s α of psychological well-being measure was 0.77 (T1) and 0.75 (T2).

### Statistical analysis

To compare the differences in variables from pre- to post-intervention among the two groups, mixed-design analysis of variance (ANOVA) was employed to assess the interaction between the two factors, i.e., across time and group (Seltman, 2012; Anderson et al., 2016). Additionally, within-group analysis was conducted via repeated-measures ANOVA to test for the main effects of time (change from pre to post) on both SKY and CTR groups. We also conducted a between-group analysis based on an independent *t*-test to assess the difference between the two groups. Correlation analysis among the seven study variables was also conducted. All analyses were performed using SPSS Version 27 software, with the significance level set at 95%. The effect size was estimated as partial eta squared (partial η^2^) for all ANOVA-based tests, while Cohen’s *d* was estimated for the independent *t*-test. As suggested by Cohen (1988), effect sizes were considered Cohen’s *d* = 0.20 as small, Cohen’s *d* = 0.50 as medium, and Cohen’s *d* = 0.80 as large.

## Results

Descriptive statistics, including means and standard deviations for all measures for SKY and CTR groups at the two time points, are presented in Table 3. A baseline comparison of the two groups for all study measures was performed. The between-group analysis based on an independent *t*-test indicated a non-significant difference between the groups at pre-test stage for stress at work [*t* (62) = 0.031, *p* = 0.975, Cohen’s *d* = 0.02], anxiety at work [*t* (62) = −0.811, *p* = 0.420, Cohen’s *d* = 0.21], thriving at work [*t* (62) = 0.550, *p* = 0.584, Cohen’s *d* = 0.13], general health [*t* (62) = 0.411, *p* = 0.682, Cohen’s *d* = 0.09], emotional well-being [*t* (62) = 0.499, *p* = 0.620, Cohen’s *d* = 0.12], and psychological well-being [*t* (62) = 1.402, *p* = 0.166, Cohen’s *d* = 0.35]. However, there was a significant difference between the two groups at pre-test stage for social well-being [*t* (62) = 2.489, *p* = 0.016, Cohen’s *d* = 0.62]. Correlations among study variables are presented in Tables 4, 5.

**Table 3:** Pre-test and post-test mean scores of both groups on all measures.

	SKY group (*n* = 33)		Control group (*n* = 31)	
Measures	Pre-test Mean (SD)	Post-test Mean (SD)	Pre-test Mean (SD)	Post-test Mean (SD)
Thriving	5.42 (0.79)	6.14 (0.65)	5.51 (0.42)	5.48 (0.42)
Emotional well-being	4.12 (0.35)	4.63 (0.64)	4.18 (0.60)	4.29 (0.64)
Social well-being	4.07 (0.16)	4.65 (0.33)	4.32 (0.57)	4.35 (0.54)
Psychological well-being	4.15 (0.28)	4.56 (0.32)	4.30 (0.53)	4.32 (0.47)
General Health	3.55 (0.47)	3.98 (0.42)	3.59 (0.48)	3.55 (0.56)
Anxiety	2.23 (0.91)	1.59 (0.37)	2.08 (0.49)	2.14 (0.53)
Stress	1.83 (0.67)	1.32 (0.61)	1.83 (0.58)	1.78 (0.53)

**Table 4:** Correlations among all study variables at T1.

	1	2	3	4	5	6	7
1. Thriving	1						
2. Emotional well-being	0.52*	1					
3. Social well-being	0.50*	0.62**	1				
4. Psychological well-being	0.62*	0.61**	0.54**	1			
5. General health	0.50*	0.31*	0.36**	0.37**	1		
6. Anxiety	−0.28*	−0.19*	−0.22*	−0.19*	−0.46**	1	
7. Stress	−0.15*	−0.26*	−0.20*	−0.15*	−0.45**	−0.23**	1

*N* = 64; **p ≤ 0.001, *p < 0.05.

**Table 5:** Correlations among all study variables at T2.

	1	2	3	4	5	6	7
1. Thriving	1						
2. Emotional well-being	0.53*	1					
3. Social well-being	0.49*	0.64**	1				
4. Psychological well-being	0.63*	0.65**	0.56**	1			
5. General health	0.50*	0.32*	0.35**	0.38**	1		
6. Anxiety	−0.30*	−0.19*	−0.23*	−0.21*	−0.45**	1	
7. Stress	−0.18*	−0.27*	−0.21*	−0.16*	−0.46**	−0.28**	1

*N* = 64; **p ≤ 0.001, *p < 0.05.

### Stress at work

**Figure 2:**
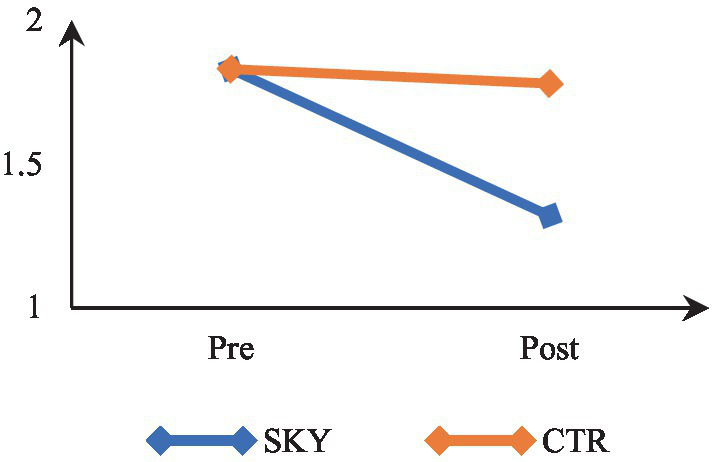
Pre-post comparison of stress.

Mixed-design ANOVA revealed a significant time by group interaction, *F*(1, 62) = 8.533, *p* = 0.005, partial η^2^ = 0.12. The results indicate that participants’ levels of stress differed significantly across the two-time points (at pre and post). There was a significant difference in stress of the two groups (SKY vs. CTR). The results of within-group analysis via repeated measures ANOVA revealed a significant change in the SKY group from the pre- to post-test, *F*(1, 32) = 18.846, *p* = 0.000, and partial η^2^ = 0.37. For the CTR group, there was a non-significant change, *F*(1, 30) = 0.217, *p* = 0.645, and partial η^2^ = 0.007. The between-group analysis based on an independent *t*-test indicated a statistically significant difference at the post-test stage [*t* (62) = 3.246, *p* = 0.002, and Cohen’s *d* = 0.80]. Figure 2 suggests that the SKY group decreased stress across the two time points. In contrast, the CTR group did not significantly change from pre to post time point.

### Anxiety at work

**Figure 3:**
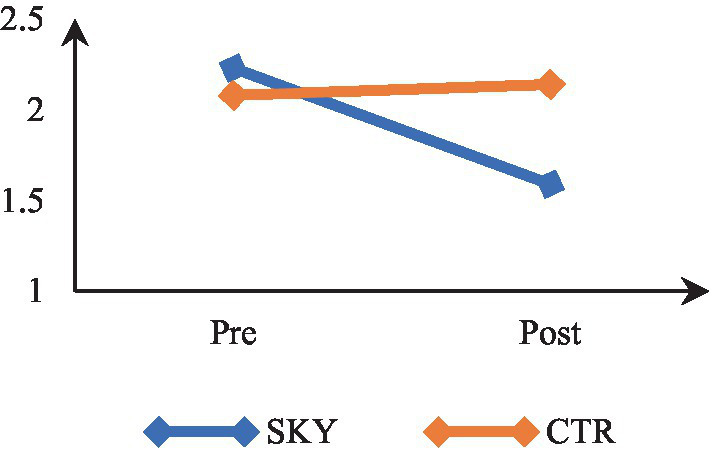
Pre-post comparision of anxiety.

Mixed-design ANOVA revealed a significant time by group interaction, *F*(1, 62) = 21.014, *p* = 0.000, and partial η^2^ = 0.25. The results indicate that participants’ anxiety levels differed significantly across the two time points (at pre and post). There was a significant difference in anxiety between the two groups (SKY vs. CTR). The results of within-group analysis via repeated measures ANOVA revealed a significant change in the SKY group from the pre to post-test, *F*(1, 32) = 21.775, *p* = 0.000, and partial η^2^ = 0.41. For the CTR group, there was a non-significant change, *F*(1, 30) = 1.013, *p* = 0.322, and partial η^2^ = 0.033. The between-group analysis based on an independent *t*-test indicated a statistically significant difference at the post-test stage [*t* (62) = 4.876, *p* = 0.000, and Cohen’s *d* = 1.20]. Figure 3 suggests that the SKY group showed a decrease in anxiety across the two time points, whereas the CTR group did not show a significant change from the pre to post time point.

### Thriving at work

**Figure 4:**
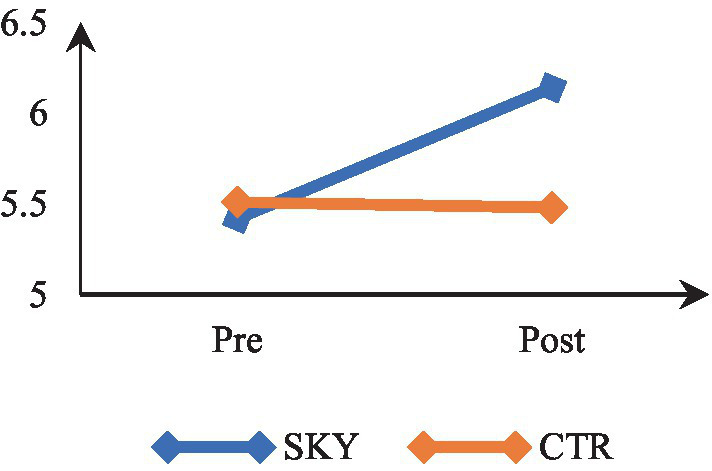
Pre-post comparision of thriving.

Mixed-design ANOVA revealed a significant time by group interaction, *F*(1, 62) = 15.150, *p* < 0.000, and partial η^2^ = 0.20. The results indicate that participants’ thriving levels differed significantly across the two time points (at pre and post). There was a significant difference in the thriving of the two groups (SKY vs. CTR). The results of within-group analysis via repeated measures ANOVA revealed a significant change in the SKY group from the pre to post-test, *F*(1, 32) = 16.997, *p* = 0.000, and partial η^2^ = 0.35. For the CTR group, there was a non-significant change, *F*(1, 30) = 0.122, *p* = 0.729, and partial η^2^ = 0.004. The between-group analysis based on an independent *t*-test indicated a statistically significant difference at the post-test stage [*t* (62) = −4.596, *p* = 0.000, and Cohen’s *d* = 1.17]. Figure 4 suggests that the SKY group showed a steady increase in thriving across the two time points, whereas the CTR group did not show a significant change from pre to post time point.

### General health

**Figure 5:**
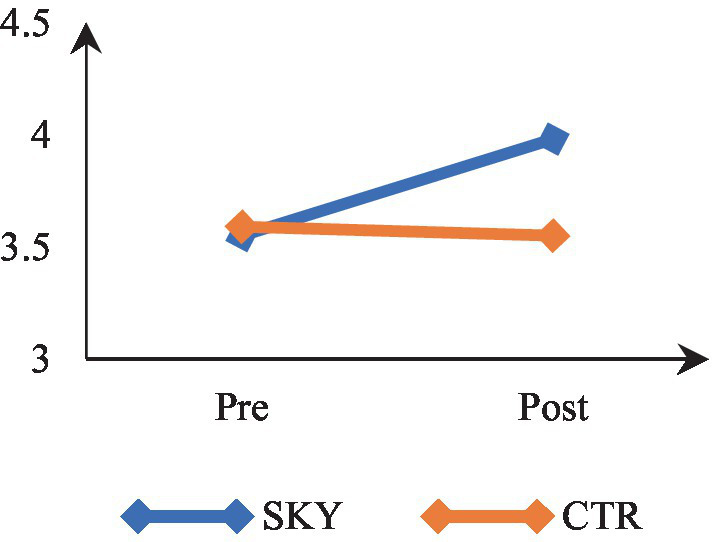
Pre-post comparision of general health.

Mixed-design ANOVA revealed a significant time by group interaction, *F*(1, 62) = 17.803, *p* = 0.000, and partial η^2^ = 0.22. The results indicate that participants’ general health levels differed significantly across the two time points (at pre and post). There was a significant difference in the general health of the two groups (SKY vs. CTR). The results of within-group analysis via repeated measures ANOVA revealed a significant change in the SKY group from the pre to post-test, *F*(1, 32) = 35.757, *p* = 0.000, and partial η^2^ = 0.53. For the CTR group, there was a non-significant change, *F*(1, 30) = 0.248, *p* = 0.622, and partial η^2^ = 0.008. The between-group analysis based on an independent *t*-test indicated a statistically significant difference at the post-test stage [*t* (62) = −3.440, *p* = 0.001, and Cohen’s *d* = 0.86]. Figure 5 suggests that the SKY group showed a steady increase in general health across the two time points. In contrast, the CTR group did not show a significant change from pre to post time point.

### Emotional well-being

**Figure 6:**
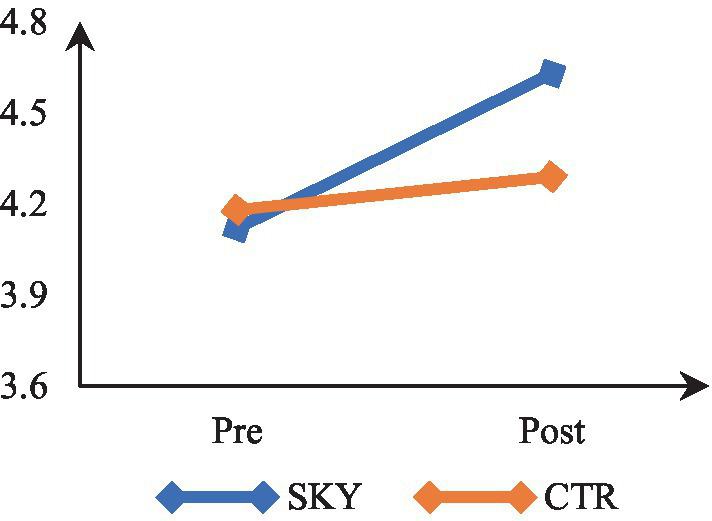
Pre-post comparision of emotional well-being.

Mixed-design ANOVA revealed a significant time by group interaction, *F*(1, 62) = 9.458, *p* = 0.003, partial η^2^ = 0.13. The results indicate that participants’ emotional well-being levels differed significantly across the two time points (at pre and post). There was a significant difference in the emotional well-being of the two groups (SKY vs. CTR). The results of within-group analysis via repeated measures ANOVA revealed a significant change in the SKY group from the pre to post-test, *F*(1, 32) = 26.865, *p* = 0.000, and partial η^2^ = 0.46. For the CTR group, there was a non-significant change, *F*(1, 30) = 1.662, *p* = 0.207, and partial η^2^ = 0.052. The between-group analysis based on an independent *t*-test indicated a statistically significant difference at the post-test stage [*t* (62) = −2.553, *p* = 0.013, and Cohen’s *d* = 0.64]. Figure 6 suggests that the SKY group showed a steady increase in emotional well-being across the two time points, whereas the CTR group did not show a significant change from pre to post time point.

### Social well-being

**Figure 7:**
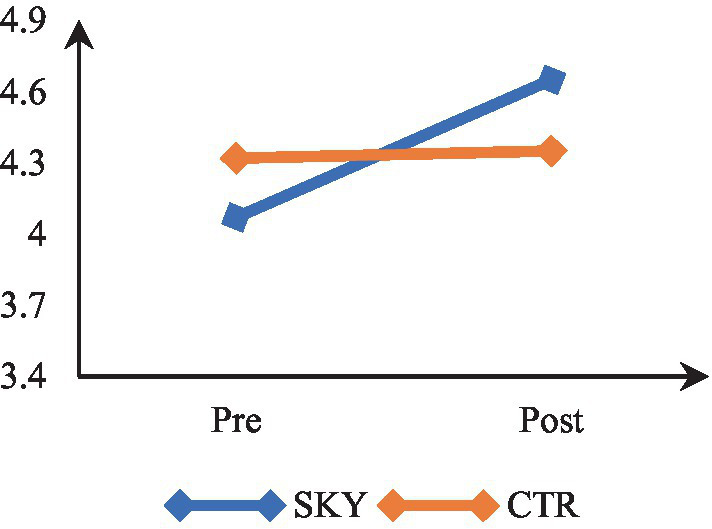
Pre-post comparision of social well-being.

Mixed-design ANOVA revealed a significant time by group interaction, *F*(1, 62) = 33.010, *p* = 0.000, and partial η^2^ = 0.35. The results indicate that participants’ social well-being levels differed significantly across the two time points (at pre and post). There was a significant difference in social well-being of the two groups (SKY vs. CTR). The results of within-group analysis via repeated measures ANOVA revealed a significant change in the SKY group from the pre to post-test, *F*(1, 32) = 100.000, *p* = 0.000, and partial η^2^ = 0.76. For the CTR group, there was a non-significant change, *F*(1, 30) = 0.111, *p* = 0.741, partial η^2^ = 0.004. The between-group analysis based on an independent *t*-test indicated a statistically significant difference at the post-test stage [*t* (62) = −2.645, *p* = 0.010, and Cohen’s *d* = 0.65]. Figure 7 suggests that the SKY group showed a steady increase in social well-being across the two time points, whereas the CTR group did not show a significant change from pre to post time point.

### Psychological well-being

**Figure 8:**
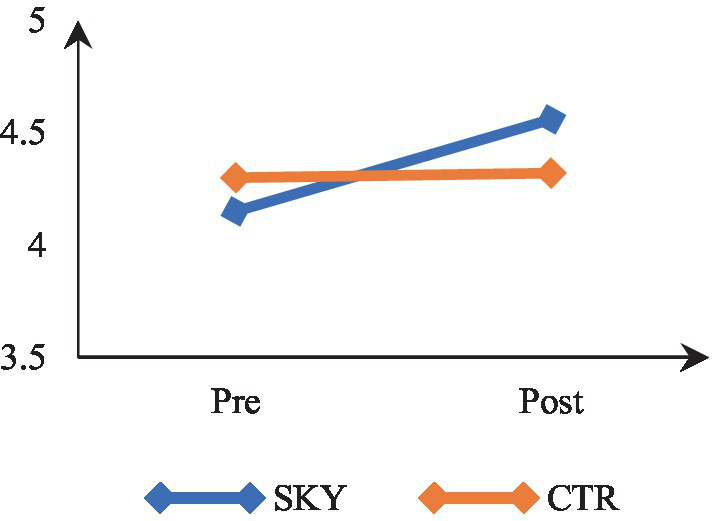
Pre-post comparision of psychological well-being.

Mixed-design ANOVA revealed a significant time by group interaction, *F*(1, 62) = 10.945, *p* = 0.002, and partial η^2^ = 0.15. The results indicate that participants’ psychological well-being levels differed significantly across the two time points (at pre and post). There was a significant difference in the psychological well-being of the two groups (SKY vs. CTR). The results of within-group analysis via repeated measures ANOVA revealed a significant change in the SKY group from the pre to post-test, *F*(1, 32) = 27.321, *p* = 0.000, and partial η^2^ = 0.46. For the CTR group, there was a non-significant change, *F*(1, 30) = 0.059, *p* = 0.809, and partial η^2^ = 0.002. The between-group analysis based on an independent *t*-test indicated a statistically significant difference at the post-test stage [*t* (62) = −2.435, *p* = 0.018, and Cohen’s *d* = 0.59]. Figure 8 suggests that the SKY group showed a steady increase in psychological well-being across the two time points, whereas the CTR group did not show a significant change from pre to post time point.

## Discussion

The study evaluated the effect of SKY intervention in alleviating stress and anxiety while promoting well-being among employees in a leading manufacturing firm. We employed a randomized-control group design. The overall results indicated that SKY had a significant effect on both negative and positive outcomes, i.e., there was a significant decline in stress and anxiety at work while a significant improvement in thriving at work, general health, emotional, social, and psychological well-being for the intervention group compared to the control group.

These findings are congruent with previous research highlighting the salubrious impact of SKY. In a sample of university students and employing a randomized controlled trial, Seppälä et al. (2020) reported that compared to the control, emotional intelligence, and mindfulness groups, SKY rendered greater benefits on the outcomes of depression, stress, mental health, positive affect, mindfulness, and social connectedness. In their study adopting convenience sampling and comparable groups, Sloan and Kanchibhotla (2023) noted that daily SKY practice was likely associated with being happier. They also reported a possible dose–response linkage of an increased frequency of SKY practice and happiness. The results of our study also concur with the prior studies that have examined the impact of SKY in the workplace. In a field experiment with a randomized wait-list control design, Mulla and Vedamuthachar (2014) found that SKY intervention significantly reduced stress and blood cortisol levels and improved life satisfaction and emotional stability in a sample of executives working at L&T (a technology, engineering, construction, and manufacturing company). Notably, breath regulation has been linked to effective neuro-cognitive function (Nakamura et al., 2018). Breathing techniques further enhance cognitive functioning and psychological flexibility related to emotional control and psychological well-being (Zaccaro et al., 2018).

In the present study, given the focus on assessing the efficacy of SKY intervention for employees’ well-being, we did not delve into determining the mechanisms of action underlying SKY. However, existing research on yogic and *pranayama* techniques provides insights into potential mechanisms. For instance, despite being a conscious process, diaphragmatic breath control unconsciously influences parasympathetic activation and conditions the autonomic nervous system, promoting a state of calmness and alertness (Jerath et al., 2006; Gard et al., 2014). The specific psychological mechanisms associated with yogic practices, including positive affect, mindfulness, self-awareness, interoceptive awareness, and self-compassion, are potential factors that could elucidate the impact of SKY on well-being and warrant further investigation (Park et al., 2021).

The current study contributes to research focusing on workplace well-being interventions. In specific ways, the findings lend support to the adoption of mind–body interventions such as yoga, meditation, and mindfulness as part of worksite stress management and wellness programs for desirable effects at the workplace (Wolever et al., 2012; Lomas et al., 2019; Kanchibhotla et al., 2022). The study reports parallel results, including stress reduction and improvements in mood, resilience, and psychological well-being (McCraty et al., 2003; Hartfiel et al., 2011).

Although the research on mindfulness-based interventions and the efficacy of physical activity programs in improving well-being across office-based workplace settings has grown (Abdin et al., 2018; Vonderlin et al., 2020), fewer studies have focused on the other mind–body-oriented approaches. Moreover, among these studies, the primary focus has been on reducing the adverse outcomes (e.g., coping with stress, anxiety management, and burnout) instead of positive outcomes (e.g., well-being and flourishing) (De Bruin et al., 2017; Cocchiara et al., 2019). This study addresses this concern by examining the impact of SKY on general health and holistic well-being. The current research also renders evidence that a breath-based SKY intervention is feasible to conduct in the workplace. Based on an organization’s schedule, commitments, and resources, SKY intervention can be delivered as a long-term, focused workshop or session-based program via in-person or online modes.

### Limitations

This study has certain limitations. Although the study had a small sample size and self-reporting, entailing the issues of demand characteristics, the findings are relevant for indicating the positive effects of SKY on psychological outcomes. Due to time and budget constraints, we could execute our field experiment at a single manufacturing firm, which limits the generalizability of findings to other sectors like information technology or finance. To address this limitation, future research may recruit participants from different firms to measure contextual variation among employees across different sectors.

Another limitation pertains to assessing the longitudinal impact and sustainability of the intervention since we collected data only before and after the SKY intervention. Future studies may include weekly practice sessions and data collection could be done again after a month or 3 months. This might provide further insights into the long-term effectiveness of SKY intervention on employees’ overall well-being.

Although we have rendered evidence supporting the efficacy of SKY in enhancing employees’ general health and well-being, the study did not explore the underlying mechanisms of action. Future research can examine the mediating variables through which SKY offers positive results. Furthermore, research may include physiological variables like blood pressure (BP), heart rate variability (HRV), or blood cortisol levels to measure the changes the breath-based intervention could have on the participants beyond the self-reported general health survey.

### Recommendations for future research

Unfortunately, due to constraints such as the unavailability of resources and permissions from authorities, the current study did not incorporate follow-up assessments beyond the immediate post-intervention period (T2). This limitation is duly acknowledged, and we recognize the importance of longitudinal studies to assess the longer-term effects of interventions. In the future, we aspire to conduct longitudinal studies to provide a more comprehensive understanding of the sustained impact of the interventions over time.

Future research could be enhanced by incorporating observer-reported measures or objective outcomes to provide a more comprehensive understanding of participants’ well-being and behaviors. Triangulating participants’ responses by gathering data on their attitudes from supervisors or peers could further enrich the study. Additionally, exploring employee performance as an objective measure in future studies could contribute valuable insights to overall well-being assessment.

Recognizing the importance of addressing potential confounding variables, we have acknowledged this as a study limitation and proposed it as part of our future research agenda. To strengthen the validity of the study, we recommend incorporating control measures, such as monitoring any health risks, high levels of blood pressure, diabetes, or heart issues, as well as tracking the consumption of any substances like alcohol or cigarettes. These additional controls could assist in identifying and accounting for potential confounding factors that might influence the outcomes of the intervention.

In the present study, a non-active control group was employed; however, we acknowledge the potential for improvement by considering including an active control group in future research. This could involve allocating participants to engage in a non-similar activity, such as diary writing, to discern the specific effects of the interventions better. We have acknowledged this limitation in the discussion section, recognizing the value of incorporating an active control group to enhance the overall rigor of the research design in subsequent studies.

## Conclusion

The therapeutic impact of yoga and SKY has explicitly been widely documented. However, in this study, we examined and indicated that the practice of rhythmic breath-based SKY intervention goes beyond the deficit-based approach of managing psychological issues of stress and anxiety and enhances thriving at work and general health, as well as the emotional, social, and psychological well-being of employees at work. It offers evidence of the efficacy of SKY as a potential, low-risk intervention to be considered by employers at the workplace for the benefit of the human capital, i.e., the organization’s workforce.

## Data availability statement

The raw data supporting the conclusions of this article will be made available by the authors, without undue reservation.

## Ethics statement

The studies involving humans were approved by Institutional Review Board of the Indian Institute of Management Indore (IRB/02/2022-23/HSS). The studies were conducted in accordance with the local legislation and institutional requirements. The participants provided their written informed consent to participate in this study.

## Author contributions

RC: Conceptualization, Data curation, Formal analysis, Investigation, Methodology, Project administration, Resources, Visualization, Writing – original draft, Writing – review & editing. CD: Conceptualization, Formal analysis, Investigation, Methodology, Validation, Writing – original draft, Writing – review & editing.
